# Supplementary material for: Intestinal pathogens override hunger-driven decision-making via immune regulation of central serotonin signaling in C. elegans
Source: Nat Commun. 2026 Feb 25;17:3144. doi: 10.1038/s41467-026-69924-w (PMC13044313; doi:10.1038/s41467-026-69924-w)
Supplement: Supplementary file 1 — Supplementary Information [file 41467_2026_69924_MOESM1_ESM.pdf]

Supplementary Information

Intestinal pathogens override hunger-driven decision-making via immune regulation of central serotonin signaling in *C. elegans*

Ying Lei, Chao Chen, Xu Zhan, Mingshu Xie, Ying Wang, Hao Li, Jiale Zhang, and Ping Liu

Supplementary Figures 1-8 and Supplementary Table 1

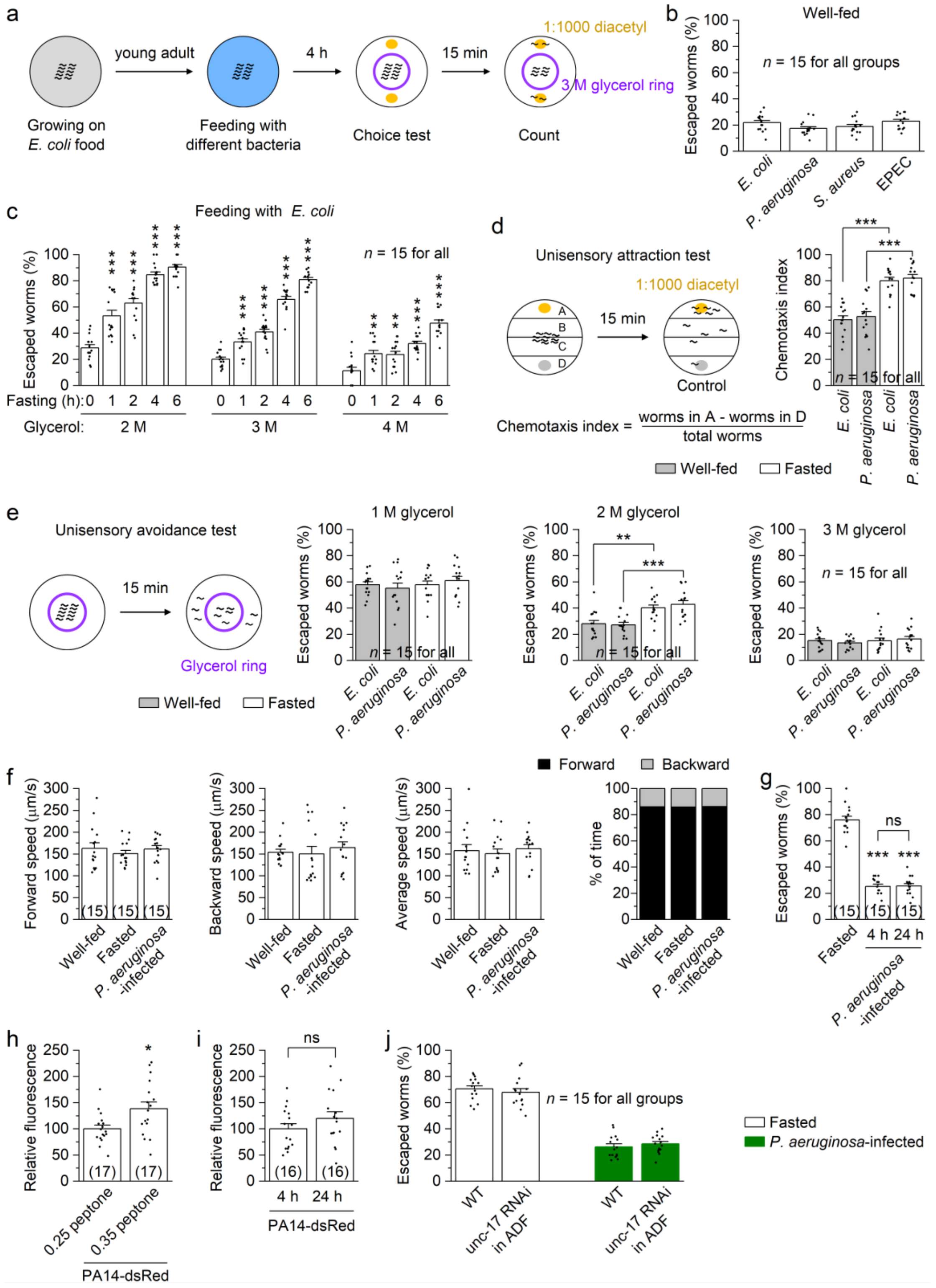

Supplementary Fig. 1. Pathogenic bacteria do not affect multisensory decision-making in well-fed *C. elegans*, and *P. aeruginosa* does not affect unisensory decision-making or locomotor kinetics in fasted *C. elegans*. a Schematic of the multisensory behavioral choice assay for well-fed *C. elegans*. **b** Escape percentages of well-fed worms fed *E. coli*, *P. aeruginosa*, *S. aureus*, or EPEC before the choice test.

p = 0.1721, 0.521, and 0.962. **c** Escape percentages of *E. coli*-fed worms fasted for the indicated time before the choice test. Assays were performed using 2, 3, or 4 M glycerol along with 1:1000 diacetyl. p = < 0.0001, 0, 0, 0, < 0.0001, 0, < 0.0001, 0, 0.0027, 0.0052, < 0.0001, and 0. **d** Chemotaxis index of well-fed or fasted worms fed *E. coli* or *P. aeruginosa* toward diacetyl in the absence of the glycerol ring. Shown are the schematic of the unisensory attraction test, equation for chemotaxis index, and the comparison. p = 0.9466, < 0.0001, and < 0.0001. **e** Escape percentages of well-fed or fasted worms fed *E. coli* or *P. aeruginosa* from the glycerol ring when diacetyl was absent. Shown are the schematic of the unisensory avoidance test and the comparison. p = 0.9313, 1, 0.5761, 0.9941, 0.004, 0.0001, 0.8872, 1, and 0.6169. **f** Forward speed, backward speed, average speed, and percentage of directional movement of well-fed, fasted, or *P. aeruginosa*-infected worms. p = 0.6462, 0.9936, 0.9761, 0.8399, 0.9021, and 0.9594. **g** Escape percentages of worms fasted for 6 hours or infected with *P. aeruginosa* for 4 or 24 hours. **h, i** Comparison of relative intestinal PA14-dsRed fluorescence intensity under the indicated conditions. p = 0.0123 (**h**) and 0.2254 (**i**). **j** Escape percentages of fasted or *P. aeruginosa*-infected worms with the indicated genotypes. ADF-specific RNAi was performed using *Psrh-142*. p = 0.4667 and 0.4446. **p < 0.01 and ***p < 0.001 (one-way ANOVA with Tukey's post hoc test for **b-g**; two-sided unpaired t test for **h-j**). ns, no significance. Brackets indicate the number of independent assays. Data are shown as means ± SEM. Source data are provided as a Source Data file.

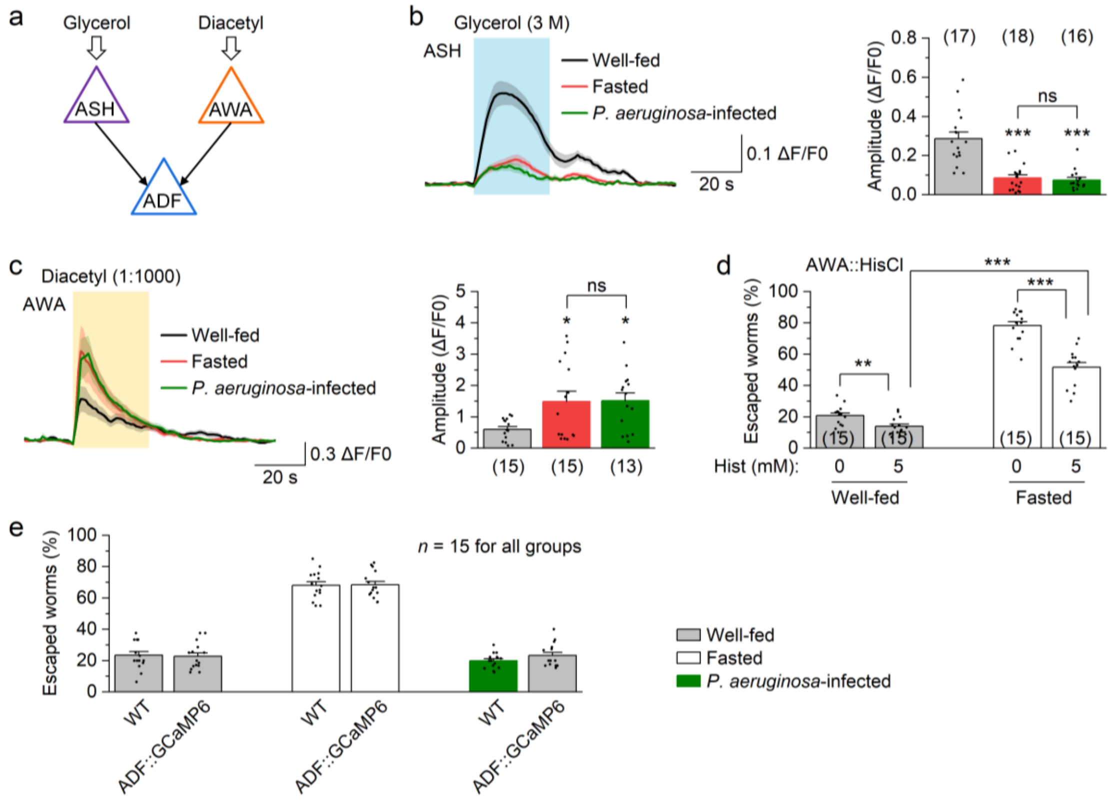

Supplementary Fig. 2. *P. aeruginosa* infection does not affect ASH responses to glycerol or AWA responses to diacetyl in fasted worms, and GCaMP6 expression in ADF does not affect decision-making. a Schematic showing that glycerol-sensing neurons ASH and diacetyl-sensing neurons AWA send synaptic input to ADF neurons. **b, c** Glycerol-evoked GCaMP6 responses in ASH (**b**) and diacetyl-evoked GCaMP6 responses in AWA (**c**) of well-fed, fasted, or *P. aeruginosa*-infected worms. Left, averaged GCaMP6 signals (solid lines, mean; shaded regions, SEM). Right, comparisons of GCaMP6 amplitudes. GCaMP6 was expressed in ASH and AWA using *Psra-6* and *Pgpa-6*, respectively. p = < 0.0001, 0.9435, and < 0.0001 (**b**), and 0.0313, 0.9964, and 0.0258 (**c**). **d** Escape percentages of well-fed or fasted worms with or without 5 mM histamine (Hist) treatment. HisCl1 was expressed in AWA using *Pgpa-6*. p = 0.0036, < 0.0001, and < 0.0001. **e** Escape percentages of worms with the indicated genotypes and conditions. WT, wild type. ADF::GCaMP6 indicates expression of GCaMP6 in ADF using *Psrh-142*. p = 0.3329, 0.9327, and 0.17. *$p < 0.05$, **$p < 0.01$, and ***$p < 0.001$ (one-way ANOVA with Tukey's post hoc test for **b**, **c**; two-sided unpaired t test for **d, e**). ns, no significance. Brackets indicate the number of animals

tested (**b**, **c**) or independent assays (**d, e**). Data are shown as means ± SEM. Source data are provided as a Source Data file.

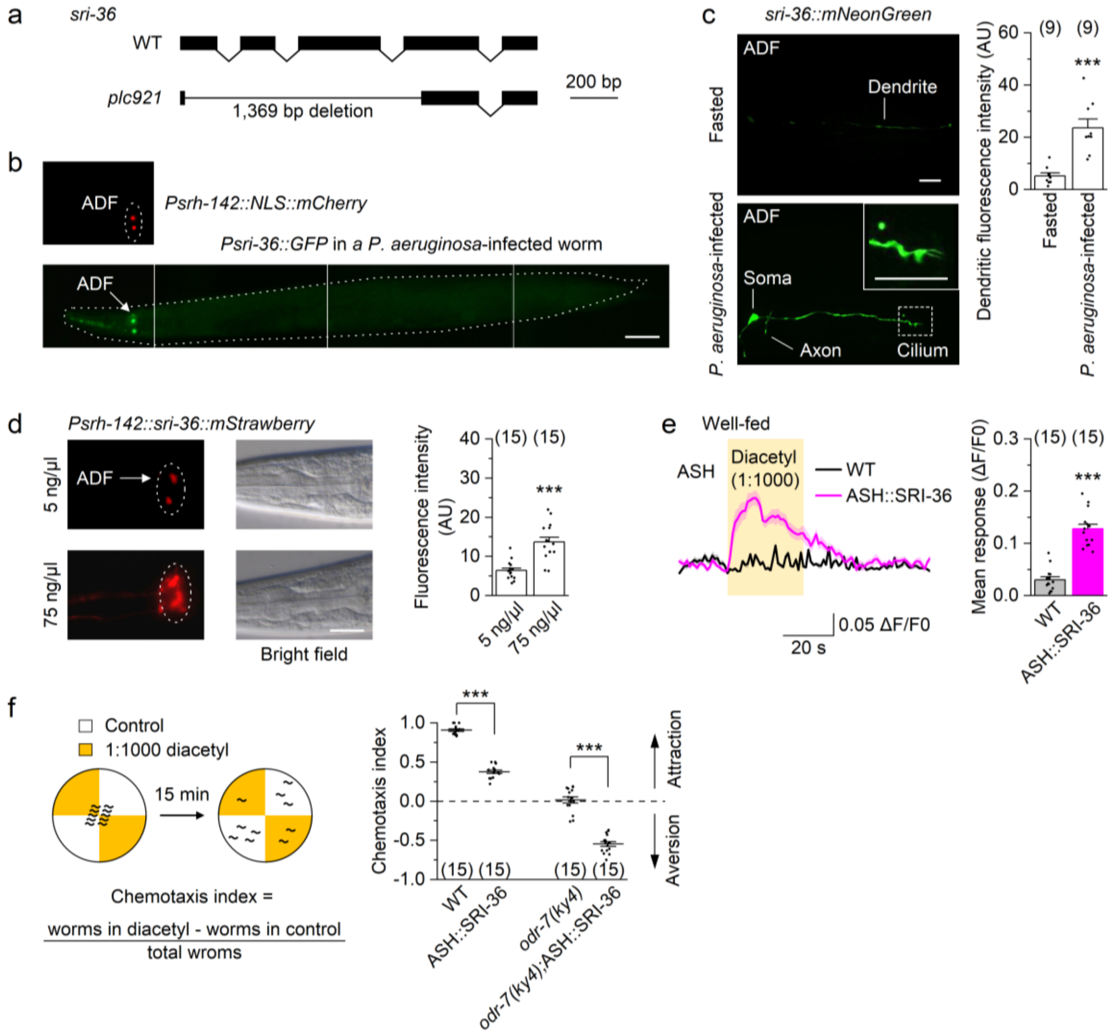

Supplementary Fig. 3. Expression pattern and subcellular localization of SRI-36, and ectopic expression of *sri-36* confers diacetyl sensitivity to ASH nociceptive neurons. a Schematic showing the deletion mutation in the *sri-36(plc921)* allele. **b** Representative whole-body image showing *Psri-36::GFP* expression in a *P. aeruginosa*-infected worm. GFP fluorescence was detected exclusively in ADF neurons. The *glo-4(ok623)* mutant background was used to eliminate non-specific intestinal autofluorescence. Scale bar, 50 µm. A similar pattern was observed in 6 animals. **c** Expression of endogenous *SRI-36::mNeonGreen* in ADF neurons of fasted or *P. aeruginosa*-infected worms. Shown are representative images and comparisons of dendritic mNeonGreen fluorescence intensity. The inset shows mNeonGreen signal in the ADF cilium of a *P. aeruginosa*-infected worm (region with dashed outline). *mNeonGreen* was fused in-frame to the C terminus of the endogenous *sri-36* locus. AU, arbitrary units. Scale bar, 20 µm. $p < 0.0001$. **d** Representative images and

comparisons of mCherry fluorescence in ADF neurons of *sri-36(plc921)* mutants injected with 5 ng/μl or 75 ng/μl *Psrh-142::sri-36::mStrawberry* plasmid. Scale bar, 20 μm. $p < 0.0001$. **e** Diacetyl-evoked GCaMP6 responses in ASH neurons of well-fed worms with the indicated genotypes. Left, averaged GCaMP6 signals (solid lines, mean; shaded regions, SEM). Right, comparisons of mean responses during the 30-s stimulation period. GCaMP6 was expressed in ASH using *Psra-6*. WT, wild type. ASH::SRI-36 indicates ectopic expression of *sri-36* in ASH using *Psra-6*. $p < 0.0001$. **f** Chemotaxis index of well-fed worms with the indicated genotypes toward diacetyl in the two-choice chemotaxis quadrant assay. Shown are the schematic of the assay, equation for chemotaxis index, and the comparison. $p < 0.0001$ and < 0.0001. ***$p < 0.001$ (two-sided unpaired *t* test). Brackets indicate the number of animals tested (**c, d, e**) or independent assays (**f**). Data are shown as means ± SEM. Source data are provided as a Source Data file.

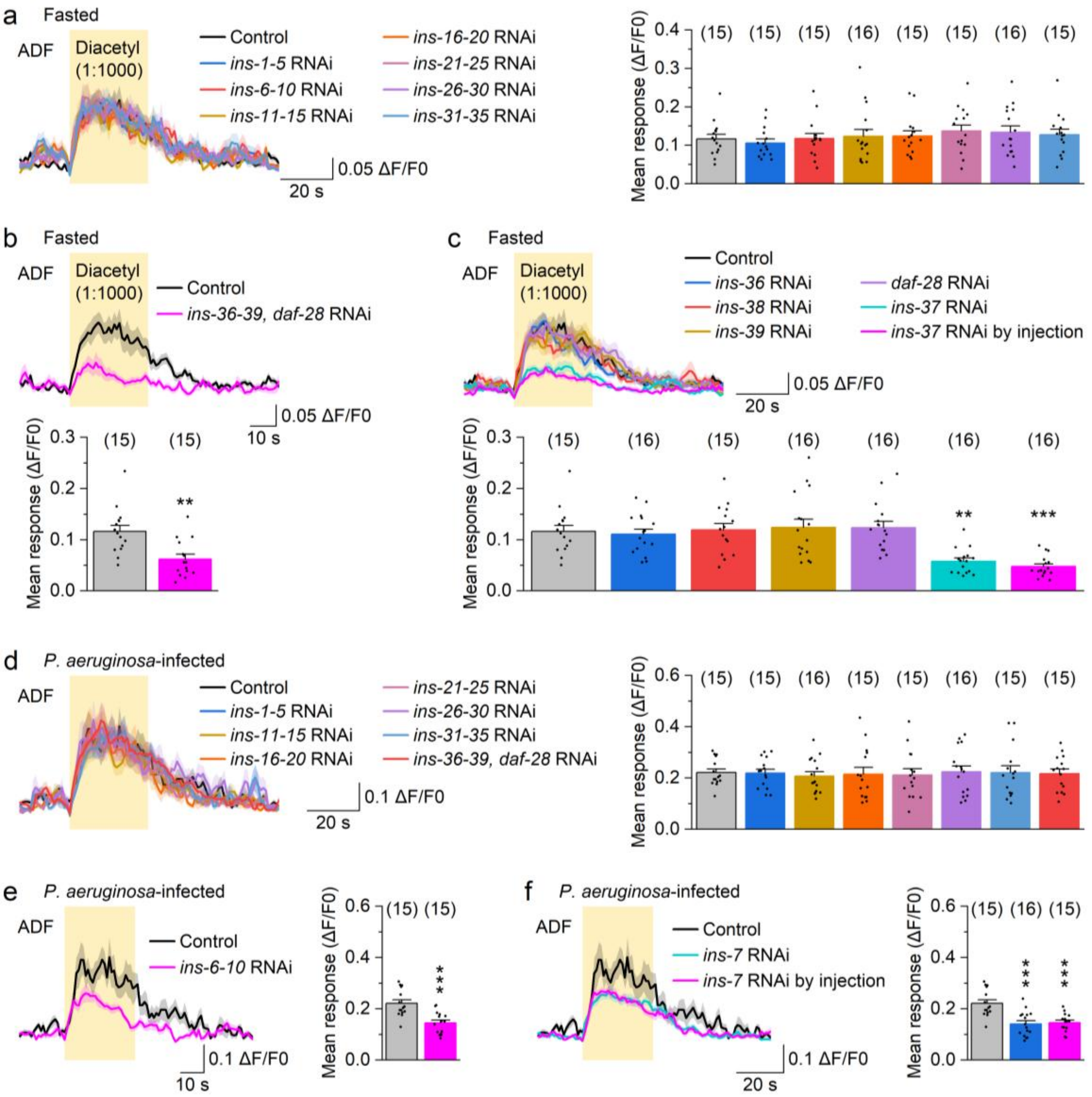

Supplementary Fig. 4. Hunger and *P. aeruginosa* infection induce the release of INS-37 and INS-7 from the intestine to regulate ADF diacetyl sensitivity. a-f Diacetyl-evoked GCaMP6 responses in ADF of worms with the indicated genotypes and conditions. Shown are averaged GCaMP6 signals (solid lines, mean; shaded areas, SEM) and comparisons of mean responses during the 30-s stimulation period. Intestine-specific RNAi of ILP genes was performed by feeding *C. elegans* strain VP303 with *E. coli* HT115 carrying either empty L4440 plasmids (controls) or L4440 plasmids containing ILP gene fragments. RNAi results for *ins-37* and *ins-7* were further confirmed by intestine-specific RNAi via plasmid injection (**c**, **f**). The same control data were used in **a-c** (fasted) and **d-f** (*P. aeruginosa*-infected), respectively. p = 0.9996, 1, 1, 0.9999, 0.9688, 0.9853, and 0.9994 (**a**), 0.0012 (**b**), 0.9999, 1, 0.9987, 0.9992, 0.0052, and 0.0005 (**c**), 1, 0.9998, 1, 1, 1, 1, and 1 (**d**), 0.0002 (**e**), and < 0.0001 and

0.0002 (**f**). **$p < 0.01$ and ***$p < 0.001$ (one-way ANOVA with Tukey's post hoc test for **a**, **c**, **d**, **f**; two-sided unpaired *t* test for **b**, **e**). Brackets indicate the number of animals tested. Data are shown as means ± SEM. Source data are provided as a Source Data file.

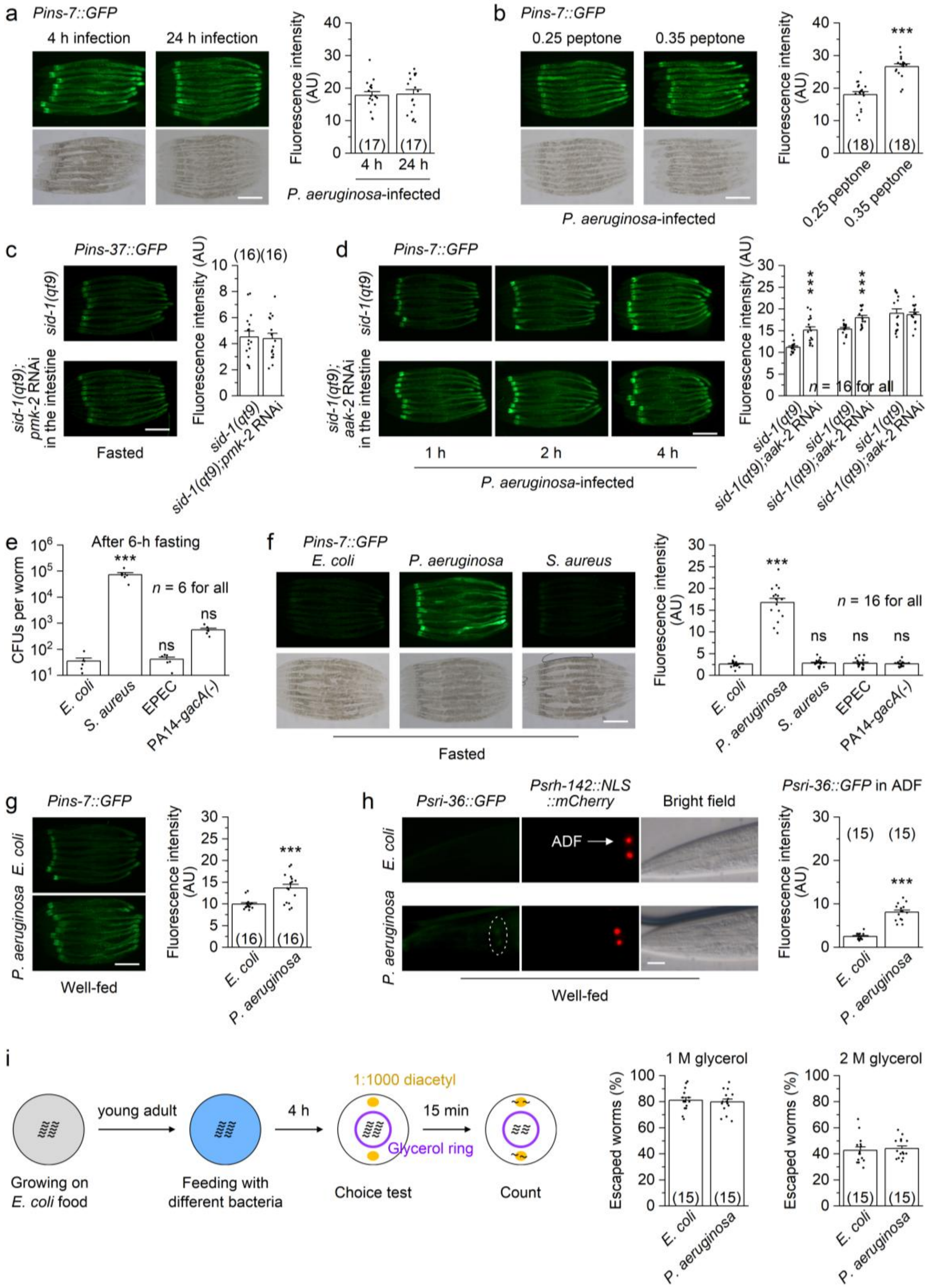

Supplementary Fig. 5 *S. aureus* colonizes the intestine but does not induce *Pins-7::GFP* expression in fasted worms, and *P. aeruginosa* activates intestinal immune signaling in well-fed worms without affecting multisensory decision-making. a, b *Pins-7::GFP* expression in *P. aeruginosa*-infected worms under the

indicated conditions. Shown are representative images and comparisons of fluorescence intensity. AU, arbitrary units. Scale bar, 100 µm. p = 0.8483 (**a**) and < 0.0001 (**b**). **c** *Pins-37::GFP* expression in fasted worms with the indicated genotypes. The *sid-1(qt9)* background was used to ensure RNAi tissue specificity. Scale bar, 100 µm. p = 0.8219. **d** *Pins-7::GFP* expression in *P. aeruginosa*-infected worms with the indicated genotypes and conditions. Scale bar, 100 µm. p = < 0.0001, 0.0003, and 0.7898. **e** CFUs of *E. coli, S. aureus,* EPEC, and PA14-*gacA(-)* after fasting. p = < 0.0001, 1, and 0.0.999. **f, g** *Pins-7::GFP* expression in fasted (**f**) and well-fed (**g**) worms fed the indicated bacteria. Scale bar, 100 µm. p = 0, 0.9971, 0.9988, and 1 (**f**), and 0.0002 (**g**). **h** *Psri-36::GFP* expression in well-fed worms fed *E. coli* or *P. aeruginosa*. Shown are representative images and comparisons of GFP fluorescence intensity. ADF neurons were labeled with *Psrh-142::NLS::mCherry*. Scale bar, 20 µm. p < 0.0001. **i** Escape percentages of well-fed worms fed *E. coli* or *P. aeruginosa*. p = 0.6955 and 0.6786. ***p < 0.001 (one-way ANOVA with Tukey's post hoc test for **e**, **f**; two-sided unpaired t test for **a-d, g-i**). n indicates the number of animals tested (**a-h**) or independent assays (**i**). Data are shown as means ± SEM. Source data are provided as a Source Data file.

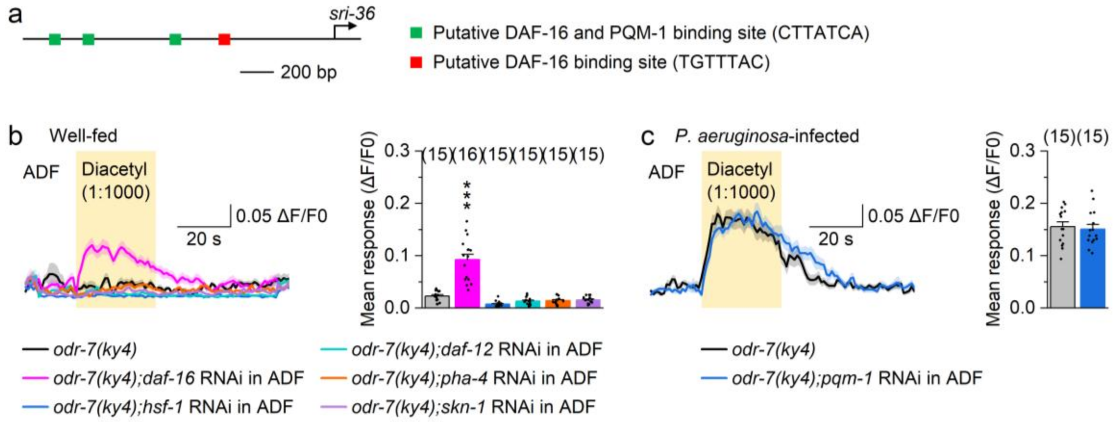

Supplementary Fig. 6. ADF-specific RNAi of *daf-16*, but not *hsf-1*, *daf-12*, *pha-4*, *skn-1*, or *pqm-1*, alters diacetyl-evoked responses in ADF neurons of *odr-7(ky4)* mutants. a Schematic showing predicted DAF-16 and PQM-1 binding sites in the upstream regulatory sequence of *sri-36*. **b, c** Diacetyl-evoked GCaMP6 responses in ADF neurons of well-fed (**b**) or *P. aeruginosa*-infected (**c**) worms with the indicated genotypes. Left, averaged GCaMP6 signals (solid lines, mean; shaded regions, SEM). Right, comparisons of mean responses during the 30-s stimulation period. ADF-specific RNAi was performed using *Psrh-142*. p = 0, 0.2016, 0.7055, 0.7936, and 0.8685 (**b**), and 0.7223 (**c**). ***$p < 0.001$ (one-way ANOVA with Tukey's post hoc test for **b**; two-sided unpaired t test for **c**). Brackets indicate the number of animals tested. Data are shown as means ± SEM. Source data are provided as a Source Data file.

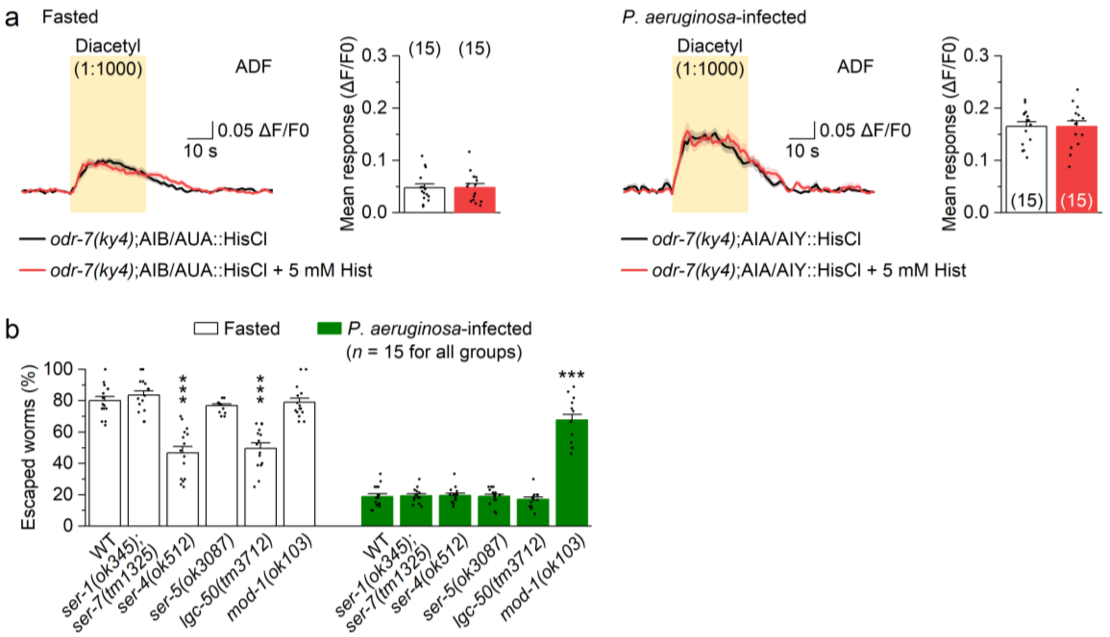

Supplementary Fig. 7. Silencing AIB/AUA or AIA/AIY did not affect ADF diacetyl sensitivity, and 5-HT receptor mutants impaired escape in fasted and infected worms. a Diacetyl-evoked GCaMP6 responses in ADF neurons of fasted or *P. aeruginosa*-infected worms with the indicated genotypes. Left, averaged GCaMP6 signals (solid lines, mean; shaded regions, SEM). Right, comparisons of mean responses during the 30-s stimulation period. Neuron-specific HisCl1 expression was performed using *Pgcy-28* (AIA), *Pttx-3* (AIY), *Pnpr-9* (AIB), and *Pflp-8* (AUA/URX). p = 0.9808 and 0.9862. **b** Escape percentages of fasted or *P. aeruginosa*-infected worms with the indicated genotypes. p = 0.9594, < 0.0001, 0.9722, < 0.0001, 0.9999, 1, 0.9999, 1, 0.9894, and 0. ***p < 0.001 (one-way ANOVA with Tukey's post hoc test for **b**; two-sided unpaired t test for **a**). Brackets indicate the number of animals tested (**a**) or independent assays (**b**). Data are shown as means ± SEM. Source data are provided as a Source Data file.

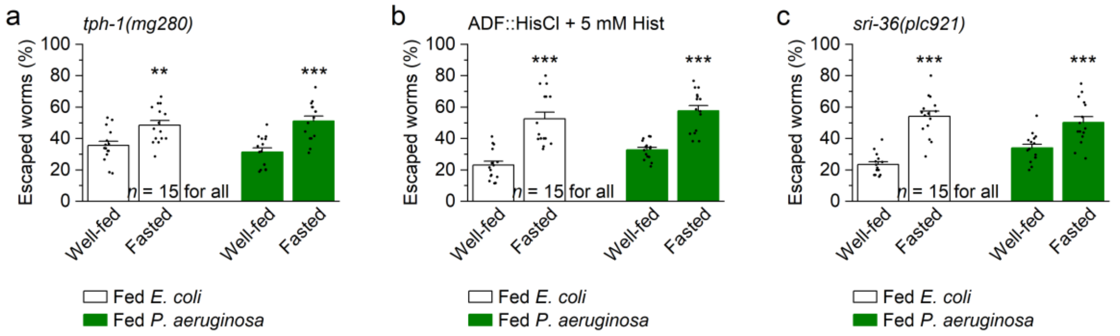

Supplementary Fig. 8. Fasting increases escape in *tph-1(mg280)* mutants, ADF-silenced worms, and *sri-36(plc921)* mutants. a-c Escape percentages of well-fed or fasted worms fed *E. coli* or *P. aeruginosa* with the indicated genotypes and conditions. ADF::HisCl indicates expression of HisCl1 in ADF using *Psrh-142*. Data for fasted worms were reused from Fig. 1h, 1j, and 3f. $p = 0.0033$ and < 0.0001 (**a**), < 0.0001 and < 0.0001 (**b**), and < 0.0001 and 0.0009 (**c**). $^{**}p < 0.01$ and $^{***}p < 0.001$ (two-sided unpaired t test). n indicates the number of independent assays. Data are shown as means ± SEM. Source data are provided as a Source Data file.

Supplementary Table 1. Primers used in this study.

Name of DNA fragment	PCR product length	**Sequence of primers** (F, forward; R, reverse)
tph-1 (cDNA)	1599 bp	F: ATGGATTCGTTGTTTCAGATG
		R: CTACAGGATGTAGTGGAGAG
ser-4 (cDNA)	1338 bp	F: ATGATCGACGAGACGCTTCTC
		R: TCAATAATCGTGAATAAGGCAC
lgc-50 (cDNA)	1476 bp	F: ATGCGATTTCTTCTTGTTCTTC
		R: TTACATGGGACGATCCATTTTC
mod-1 (gDNA)	2463 bp	F: ATGAAGTTTATTCCTGAAATCAC
		R: TCACTGATAGTTTTGATCGAAAG
daf-16 (cDNA)	1530 bp	F: ATGATGGAGATGCTGGTAGATC
		R: TTACAAATCAAAATGAATATGCTGCCC
sri-36 (gDNA)	1812 bp	F: ATGACGATAAACTTCACCACCCCTGTC
		R: TTAAAACATGGCTATATTTCTAACTCC
odr-3 (cDNA)	1071 bp	F: ATGGGCTCATGCCAGAGCAATG
		R: TTACATCATTCCTGCTTTTTG
ocr-2 (cDNA)	2703 bp	F: ATGGGTTCCTCATCTTCAACCCAATC
		R: TCAGTGAGCAGCACCATTTCCATTCTG
osm-9 (cDNA)	2814 bp	F: ATGGGCGGTGGAAGTTCGCGAAAC
		R: TCATTCGCTTTTGTCATTTGTCGGC
ins-7 (cDNA)	351 bp	F: ATGTATAAAGTACATTATTTTC
		R: TTAAGGACAGCACTGTTTTCG
hid-1 RNAi	472 bp	F: ATGGGTGCTCAGGGTAGCAG
		R: CGATTGTGGAAAGATCGTCG
nol-6 RNAi	477 bp	F: ATGGAGACAGACGGAGATGTG
		R: GAAAATTGGGTCATCTCTGTG
daf-2 RNAi	558 bp	F: CGACGAAATACTGGTGCATC
		R: TCAGACAAGTGGATGATGCTC
ins-7 RNAi	276 bp	F: ATGCCACCAATAATTTTGG
		R: TTCACGGCAACATTTTGATG
ins-37 RNAi	406 bp	F: ATGGCTGCTTTCCTGCCAATTG
		R: TTGTTCGGGCACATTGCTTC
daf-16 RNAi	446 bp	F: ATCATGGGTTGGCGAATCGG
		R: CCATAGTATCCATCAGTGGC
nsy-1 RNAi	558 bp	F: GTCAGCGATTCCAGGAAATGC
		R: TCGCGAGAATGTGCTGATGC
sek-1 RNAi	478 bp	F: GAATCTGGAAGACAGATGTC
		R: ATCTTGACTTGTCCATGTCG
sri-36 RNAi	793 bp	F: CCTATGCACCATAACGAATATC
		R: CATGGCTATATTTCTAACTCC

aak-2 RNAi	543 bp	F: TCACACCAACTCTCGACAAC<br>R: GAGCCAGTGTTCCAATCAATG
ser-4 RNAi	573 bp	F: ATTTCAGTTGACGTGCTCGTC<br>R: CATTGTTCGTTCTTCTTCGG
mod-1 RNAi	554 bp	F: TTTGTACACTCTACACAGGC<br>R: AATGTGACAGCTTCTTCCATCC
lgc-50 RNAi	591 bp	F: GGCAAGGATAACACTTGGTGTC<br>R: GAACACCGATCCACTTGTACAC
Pges-1	3228 bp	F: GGAATCCGCCAAATTGTCGAC<br>R: CTGAAACTGTAAGACGCAG
Pceh-2	1567 bp	F: GGAATCGAACCAAAGCTTAAATC<br>R: TTTCACTCCGAATATTAGAAAAAATAAG
Psrh-142	1452 bp	F: GGTCGCGAGCTTTGATTTCCTTC<br>R: ATTGGCAAAAAGAAAAAAGAGGTGC
Podr-2	2650 bp	F: GTTAATTGAACTGATACTAGTCAGC<br>R: TTCTGTCTGAAATATAAATGTTCCG
Pser-2prom2	4733 bp	F: GTCTGGTAAGTTGAACATGGAGTC<br>R: TTTTGCAAATTACTTGAGGCTGC
Pflp-8	3178 bp	F: GCAATGAGTGCTCAAATGGAG<br>R: TTTCTACTTGAAAAGTGTGGACTGA
Pgcy-36	1029 bp	F: GTAGTTCGGATTCTGAAAAGGCC<br>R: TGTTGGGTAGCCCTTGTTTG
Pglr-3	2088 bp	F: AGAGTTGAACTCATTAACCTC<br>R: ATGTTAATAGCAAATATTG
Psra-6	2409 bp	F: TCTGAGGTGCATTTGCGA<br>R: CGGCAAAATCTGAAATAATAAATA
Pclh-3	3390 bp	F: CAAATCAAGTGACGCAATCTGACTCGC<br>R: ACCAATACCCAAACTTTTGGAATCCTCG
Pgcy-28	2841 bp	F: TACAATTGTAGTGAGCTTCG<br>R: TTCGCACTCATCTCACCATTC
Pttx-3	3179 bp	F: GTACTCGCATAGTAAATAAG<br>R: TTGAAAAGTAGGAAGCATTG
Pnpr-9	1975 bp	F: GTTTCAATTACCAGTCTTTC<br>R: GGAAGTAGCTCTAAAATTAC
