# Supplementary material for: Intestinal pathogens override hunger-driven decision-making via immune regulation of central serotonin signaling in C. elegans
Source: Nat Commun. 2026 Feb 25;17:3144. doi: 10.1038/s41467-026-69924-w (PMC13044313; doi:10.1038/s41467-026-69924-w)
Supplement: Supplementary file 2 — Description of Additional Supplementary Files [file 41467_2026_69924_MOESM2_ESM.pdf]

Description of Additional Supplementary Files

Supplementary Data 1: List of Bacterial and C. elegans strains
