# Supplementary material for: Intestinal pathogens override hunger-driven decision-making via immune regulation of central serotonin signaling in C. elegans
Source: Nat Commun. 2026 Feb 25;17:3144. doi: 10.1038/s41467-026-69924-w (PMC13044313; doi:10.1038/s41467-026-69924-w)
Supplement: Supplementary file 4 — Reporting Summary [file 41467_2026_69924_MOESM4_ESM.pdf]

nature portfolio

Corresponding author(s): Ping Liu

Last updated by author(s): Jan 12, 2026

Reporting Summary

Nature Portfolio wishes to improve the reproducibility of the work that we publish. This form provides structure for consistency and transparency in reporting. For further information on Nature Portfolio policies, see our Editorial Policies and the Editorial Policy Checklist.

Statistics

For all statistical analyses, confirm that the following items are present in the figure legend, table legend, main text, or Methods section.

n/a	Confirmed	
☐	☒	The exact sample size (*n*) for each experimental group/condition, given as a discrete number and unit of measurement
☐	☒	A statement on whether measurements were taken from distinct samples or whether the same sample was measured repeatedly
☐	☒	The statistical test(s) used AND whether they are one- or two-sided<br>*Only common tests should be described solely by name; describe more complex techniques in the Methods section.*
☒	☐	A description of all covariates tested
☒	☐	A description of any assumptions or corrections, such as tests of normality and adjustment for multiple comparisons
☐	☒	A full description of the statistical parameters including central tendency (e.g. means) or other basic estimates (e.g. regression coefficient) AND variation (e.g. standard deviation) or associated estimates of uncertainty (e.g. confidence intervals)
☐	☒	For null hypothesis testing, the test statistic (e.g. *F*, *t*, *r*) with confidence intervals, effect sizes, degrees of freedom and *P* value noted<br>*Give P values as exact values whenever suitable.*
☒	☐	For Bayesian analysis, information on the choice of priors and Markov chain Monte Carlo settings
☒	☐	For hierarchical and complex designs, identification of the appropriate level for tests and full reporting of outcomes
☒	☐	Estimates of effect sizes (e.g. Cohen's *d*, Pearson's *r*), indicating how they were calculated

Our web collection on statistics for biologists contains articles on many of the points above.

Software and code

Policy information about availability of computer code

Data collection	MShot Image Analysis System (version 1.1) was used for imaging collection; Track-A-Worm automated tracking system (version 2.0) was used for locomotion tests.
Data analysis	Fluorescence, calcium, and pHluorin imaging data were analyzed using ImageJ (version 1.50i); Locomotion behavior was analyzed using Track-A-Worm (version 2.0); Statistical analyses and data graphing were performed using Origin 2019 (OriginLab).

For manuscripts utilizing custom algorithms or software that are central to the research but not yet described in published literature, software must be made available to editors and reviewers. We strongly encourage code deposition in a community repository (e.g. GitHub). See the Nature Portfolio guidelines for submitting code & software for further information.

Data

Policy information about availability of data

All manuscripts must include a data availability statement. This statement should provide the following information, where applicable:

- Accession codes, unique identifiers, or web links for publicly available datasets
- A description of any restrictions on data availability
- For clinical datasets or third party data, please ensure that the statement adheres to our policy

All data generated or analyzed during this study are included in this published article and its supplementary information files. Source data are provided with this paper.

Research involving human participants, their data, or biological material

Policy information about studies with human participants or human data. See also policy information about sex, gender (identity/presentation), and sexual orientation and race, ethnicity and racism.

Reporting on sex and gender	All experiments were performed using young adult hermaphrodites.
Reporting on race, ethnicity, or other socially relevant groupings	not applicable
Population characteristics	not applicable
Recruitment	not applicable
Ethics oversight	The study uses the nematode C. elegans, which does not require Ethical approval.

Note that full information on the approval of the study protocol must also be provided in the manuscript.

Field-specific reporting

Please select the one below that is the best fit for your research. If you are not sure, read the appropriate sections before making your selection.

☒ Life sciences ☐ Behavioural & social sciences ☐ Ecological, evolutionary & environmental sciences

For a reference copy of the document with all sections, see nature.com/documents/nr-reporting-summary-flat.pdf

Life sciences study design

All studies must disclose on these points even when the disclosure is negative.

Sample size	Sample sizes for imaging, locomotion, and behavioral assays were determined based on previous studies using similar experimental paradigms, including our previous work. No statistical methods were used to predetermine sample sizes. Instead, sample sizes were chosen to ensure adequate power to detect biological differences, and are consistent with those commonly used in the field.
Data exclusions	No data were excluded from the analyses.
Replication	Replication was achieved using different cells, different animals, or independent assays. All experiments were performed on at least two separate days. For calcium imaging and locomotion analyses, n denotes the number of animals tested, whereas for behavioral choice assays, n denotes the number of independent assays. We are not aware of any results that could not be reproduced under the described experimental conditions.
Randomization	For each genotype, animals grown on the same plate were randomly allocated to different conditions. For individual assays, animals were randomly selected. For population assays, animals from a plate were washed off with M9 buffer, and adequate amount of buffer containing animals was placed onto each choice plate.
Blinding	Blinding was not applied in this study. Locomotion assays were performed using the automated Track-A-Worm system, which minimizes experimenter involvement during data acquisition and analysis. For imaging and behavioral assays, most animals were readily identifiable based on transgenic markers, visible phenotypes, or bacterial treatment conditions.

Reporting for specific materials, systems and methods

We require information from authors about some types of materials, experimental systems and methods used in many studies. Here, indicate whether each material, system or method listed is relevant to your study. If you are not sure if a list item applies to your research, read the appropriate section before selecting a response.

Materials & experimental systems

n/a	Involved in the study
☒	☐ Antibodies
☒	☐ Eukaryotic cell lines
☒	☐ Palaeontology and archaeology
☐	☒ Animals and other organisms
☒	☐ Clinical data
☒	☐ Dual use research of concern
☒	☐ Plants

Methods

n/a	Involved in the study
☒	☐ ChIP-seq
☒	☐ Flow cytometry
☒	☐ MRI-based neuroimaging

Animals and other research organisms

Policy information about studies involving animals; ARRIVE guidelines recommended for reporting animal research, and Sex and Gender in Research

Laboratory animals

The following C. elegans strains were used:
N2 (Bristol) wild type
CB1112 cat-2(e1112)
MT15434 tph-1(mg280)
MT8944 mod-5(n822)
PLX921 sri-36(plc921)
DA2109 ser-7(tm1325);ser-1(ok345)
RB745 ser-4(ok512)
RB2277 ser-5(ok3087)
T20B12.9 lgc-50(tm3712)
MT9668 mod-1(ok103)
QZ126 ins-7(tm2001)
HC196 sid-1(qt9)
PLX812 xyhEx812[Pges-1::nol-6 RNAi, Pmyo-2::GFP]
PLX516 xyhEx516[Psrh-142::tph-1 cDNA, Pmyo-2::mStrawberry]
PLX815 xyhEx815[Pceh-2::tph-1 cDNA, Pmyo-2::mStrawberry]
PLX1130 xyhEx1130[Psrh-142::unc-17 RNAi, Pmyo-2::GFP]
PLX446 xyhEx446[Psrh-142::HisCl1::SL2::GCaMP6s, Punc-122::GFP]
PLX577 unc-13(e51);xyhEx446[Psrh-142::HisCl1::SL2::GCaMP6s, Punc-122::GFP]
PLX578 unc-31(e169);xyhEx446[Psrh-142::HisCl1::SL2::GCaMP6s, Punc-122::GFP]
PLX623 odr-7(ky4);xyhEx446[Psrh-142::HisCl1::SL2::GCaMP6s, Punc-122::GFP]
PLX642 odr-7(ky4);unc-13(e51);xyhEx446[Psrh-142::HisCl1::SL2::GCaMP6s, Punc-122::GFP]
PLX782 odr-3(n2150);odr-7(ky4);xyhEx446[Psrh-142::HisCl1::SL2::GCaMP6s, Punc-122::GFP]
PLX799 ocr-2(ak47);odr-7(ky4);xyhEx446[Psrh-142::HisCl1::SL2::GCaMP6s, Punc-122::GFP]
PLX716 osm-9(ok1677);odr-7(ky4);xyhEx446[Psrh-142::HisCl1::SL2::GCaMP6s, Punc-122::GFP]
PLX962 odr-3(n2150);odr-7(ky4);xyhEx446[Psrh-142::HisCl1::SL2::GCaMP6s, Punc-122::GFP];xyhEx962[Psrh-142::odr-3 cDNA, Pmyo-3::mStrawberry]
PLX961 ocr-2(ak47);odr-7(ky4);xyhEx446[Psrh-142::HisCl1::SL2::GCaMP6s, Punc-122::GFP];xyhEx961[Psrh-142::ocr-2 cDNA, Pmyo-3::mStrawberry]
PLX957 osm-9(ok1677);odr-7(ky4);xyhEx446[Psrh-142::HisCl1::SL2::GCaMP6s, Punc-122::GFP];xyhEx957[Psrh-142::osm-9 cDNA, Pmyo-3::mStrawberry]
PLX640 odr-10(ky32);unc-13(e51);xyhEx446[Psrh-142::HisCl1::SL2::GCaMP6s, Punc-122::GFP]
PLX735 odr-7(ky4);xyhEx446[Psrh-142::HisCl1::SL2::GCaMP6s, Punc-122::GFP];xyhEx735[Psrh-142::sri-36 RNAi, Pmyo-2::mStrawberry]
PLX809 odr-7(ky4);xyhEx809[Psrh-142::srr-6 RNAi, Pmyo-3::mStrawberry];xyhEx446[Psrh-142::HisCl1::SL2::GCaMP6s, Punc-122::GFP]
PLX787 odr-7(ky4);xyhEx446[Psrh-142::HisCl1::SL2::GCaMP6s, Punc-122::GFP];xyhEx787[Psrh-142::str-163 RNAi, Pmyo-3::mStrawberry]
PLX1002 odr-7(ky4);xyhEx446[Psrh-142::HisCl1::SL2::GCaMP6s, Punc-122::GFP];xyhEx1002[Psrh-142::srap-1 RNAi, Pmyo-3::mStrawberry]
PLX970 sri-36(plc921);odr-7(ky4);xyhEx446[Psrh-142::HisCl1::SL2::GCaMP6s, Punc-122::GFP]
PLX971 sri-36(plc921);odr-7(ky4);xyhEx446[Psrh-142::HisCl1::SL2::GCaMP6s, Punc-122::GFP];xyhEx971[Psrh-142::sri-36 gDNA, Pmyo-3::mStrawberry]
PLX1105 sri-36(plc921);odr-7(ky4);xyhEx446[Psrh-142::HisCl1::SL2::GCaMP6, Punc-122::GFP];yhEx1104[Psrh-142::sri-36 gDNA::mStrawberry (5ng/ul), Punc-122::dsRed]
PLX1107 sri-36(plc921);odr-7(ky4);xyhEx446[Psrh-142::HisCl1::SL2::GCaMP6, Punc-122::GFP];yhEx1106[Psrh-142::sri-36 gDNA::mStrawberry (75ng/ul), Punc-122::dsRed]
PLX821 odr-7(ky4);xyhEx821[Psrh-142::sri-36 RNAi, Pmyo-3::mStrawberry];xyhEx446[Psrh-142::HisCl1::SL2::GCaMP6s, Punc-122::GFP]
PLX179 xyhEx821[Psrh-142::sri-36 RNAi, Pmyo-3::mStrawberry]
PLX991 sri-36(plc921);xyhEx971[Psrh-142::sri-36 gDNA, Pmyo-3::mStrawberry]
PLX1104 sri-36(plc921);yhEx1104[Psrh-142::sri-36 gDNA::mStrawberry (5ng/ul), Punc-122::dsRed]
PLX1106 sri-36(plc921);yhEx1106[Psrh-142::sri-36 gDNA::mStrawberry (75ng/ul), Punc-122::dsRed]
PLX498 xyhEx498[Psri-36::GFP, Psrh-142::NLS::mCherry, Punc-122::GFP]

PLX1100 plc1100[sri-36::mNeonGreen]
PLX1129 glo-4(ok623);xyhEx1129[Psri-36::GFP, Psrh-142::NLS::mCherry]
PLX1121 sid-1(qt9);xyhEx498[Psri-36::GFP, Psrh-142::NLS::mCherry, Punc-122::GFP]
PLX1103 plc1100[sri-36::mNeonGreen];yhEx1103[Psrh-142::mStrawberry, Punc-122::dsRed]
PLX1001 xyhEx1001[Psrh-142::sri-36 gDNA::GFP, Pmyo-3::mStrawberry]
PLX1073 sid-1(qt9);odr-7(ky4);xyhEx446[Psrh-142::HisCl1::SL2::GCaMP6, Punc-122::GFP]
PLX951 aak-2(ok524);odr-7(ky4);xyhEx446[Psrh-142::HisCl1::SL2::GCaMP6s, Punc-122::GFP]
PLX990 aak-2(ok524);odr-7(ky4);xyhEx446[Psrh-142::HisCl1::SL2::GCaMP6s, Punc-122::GFP];xyhEx990[Pges-1::aak-2 cDNA, Pmyo-3::mStrawberry]
PLX827 odr-7(ky4);xyhEx827[Pges-1::aak-2 RNAi, Pmyo-3::mStrawberry];xyhEx446[Psrh-142::HisCl1::SL2::GCaMP6s, Punc-122::GFP]
PLX1066 sid-1(qt9);odr-7(ky4);xyhEx827[Pges-1::aak-2 RNAi, Pmyo-3::mStrawberry];xyhEx446[Psrh-142::HisCl1::SL2::GCaMP6, Punc-122::GFP]
PLX802 odr-7(ky4);xyhEx802[Pges-1::daf-16 RNAi, Pmyo-3::mStrawberry];xyhEx446[Psrh-142::HisCl1::SL2::GCaMP6s, Punc-122::GFP]
PLX1065 sid-1(qt9);odr-7(ky4);xyhEx802[Pges-1::daf-16 RNAi, Pmyo-3::mStrawberry];xyhEx446[Psrh-142::HisCl1::SL2::GCaMP6, Punc-122::GFP]
PLX940 aak-2(ok524);odr-7(ky4);xyhEx446[Psrh-142::HisCl1::SL2::GCaMP6s, Punc-122::GFP];xyhEx940[Pges-1:daf-16aAM::EGFP, Pmyo-3::mStrawberry]
PLX896 zls356[Pdaf-16::daf-16a/b::GFP, rol-6(su1006)];xyhEx896[Pges-1::NLS::mCherry,Pmyo-2::mStrawberry]
PLX1004 zls356[Pdaf-16::daf-16a/b::GFP, rol-6(su1006)];xyhEx1004[Pges-1::NLS::mCherry, Pges-1::aak-2 RNAi, Pmyo-2::mStrawberry]
PLX1005 zls356[Pdaf-16::daf-16a/b::GFP, rol-6(su1006)];xyhEx1005[Pges-1::NLS::mCherry, Pges-1::pmk-2 RNAi, Pmyo-2::mStrawberry]
PLX1110 sid-1(qt9);zls356[Pdaf-16::daf-16a/b::GFP, rol-6(su1006)];xyhEx896[Pges-1::NLS::mCherry,Pmyo-2::mStrawberry]
PLX1111 sid-1(qt9);zls356[Pdaf-16::daf-16a/b::GFP, rol-6(su1006)];xyhEx1004[Pges-1::NLS::mCherry, Pges-1::aak-2 RNAi, Pmyo-2::mStrawberry]
PLX1112 sid-1(qt9);zls356[Pdaf-16::daf-16a/b::GFP, rol-6(su1006)];xyhEx1005[Pges-1::NLS::mCherry, Pges-1::pmk-2 RNAi, Pmyo-2::mStrawberry]
PLX916 nsy-1(ag3);odr-7(ky4);xyhEx446[Psrh-142::HisCl1::SL2::GCaMP6s, Punc-122::GFP]
PLX800 odr-7(ky4);xyhEx800[Pges-1::nsy-1 RNAi, Pmyo-3::mStrawberry];xyhEx446[Psrh-142::HisCl1::SL2::GCaMP6s, Punc-122::GFP]
PLX1097 sid-1(qt9);odr-7(ky4);xyhEx446[Psrh-142::HisCl1::SL2::GCaMP6, Punc-122::GFP];xyhEx800[Pges-1(3.2kb)::nsy-1 RNAi, Pmyo-3::mStrawberry]
PLX956 sek-1(km4);odr-7(ky4);xyhEx446[Psrh-142::HisCl1::SL2::GCaMP6s, Punc-122::GFP]
PLX807 odr-7(ky4);xyhEx807[Pges-1::sek-1 RNAi, Pmyo-3::mStrawberry];xyhEx446[Psrh-142::HisCl1::SL2::GCaMP6s, Punc-122::GFP]
PLX1098 sid-1(qt9);odr-7(ky4);xyhEx446[Psrh-142::HisCl1::SL2::GCaMP6, Punc-122::GFP];xyhEx807[Pges-1(3.2kb)::sek-1 RNAi, Pmyo-3::mStrawberry]
PLX909 pmk-1(km25);odr-7(ky4);xyhEx446[Psrh-142::HisCl1::SL2::GCaMP6s, Punc-122::GFP]
PLX709 odr-7(ky4);xyhEx446[Psrh-142::HisCl1::SL2::GCaMP6s, Punc-122::GFP];xyhEx709[Pges-1::pmk-2 RNAi, Pmyo-3::mStrawberry]
PLX1063 sid-1(qt9);odr-7(ky4);xyhEx446[Psrh-142::HisCl1::SL2::GCaMP6, Punc-122::GFP];xyhEx709[Pges-1::pmk-2 RNAi, Pmyo-3::mStrawberry]
PLX910 pmk-3(ok169);odr-7(ky4);xyhEx446[Psrh-142::HisCl1::SL2::GCaMP6s, Punc-122::GFP]
PLX939 odr-7(ky4);xyhEx446[Psrh-142::HisCl1::SL2::GCaMP6s, Punc-122::GFP];xyhEx939[Pges-1:daf-16aAM::EGFP, Pmyo-3::mStrawberry]
PLX941 odr-7(ky4);xyhEx446[Psrh-142::HisCl1::SL2::GCaMP6s, Punc-122::GFP];xyhEx941[Pges-1::pmk-2 RNAi, Pges-1:daf-16aAM::EGFP, Pmyo-3::mStrawberry]
PLX1074 sid-1(qt9);yxIs13[Pins-7::GFP, Punc-122::dsRed];xyhEx941[Pges-1::pmk-2 RNAi, Pges-1::daf-16 cDNAaAM::EGFP, Pmyo-3::mStrawberry]
PLX1006 xyhEx498[Psri-36::GFP, Psrh-142::NLS::mCherry, Punc-122::GFP];xyhEx1006[Pges-1::aak-2 RNAi, Punc-122::dsRed]
PLX1089 sid-1(qt9);xyhEx498[Psri-36::GFP, Psrh-142::NLS::mCherry, Punc-122::GFP];xyhEx1006[Pges-1::aak-2 RNAi, Punc-122::dsRed]
PLX1007 xyhEx498[Psri-36::GFP, Psrh-142::NLS::mCherry, Punc-122::GFP];xyhEx1007[Pges-1::daf-16 RNAi, Punc-122::dsRed]
PLX1090 sid-1(qt9);xyhEx498[Psri-36::GFP, Psrh-142::NLS::mCherry, Punc-122::GFP];xyhEx1007[Pges-1::daf-16 RNAi, Punc-122::dsRed]
PLX1008 xyhEx498[Psri-36::GFP, Psrh-142::NLS::mCherry, Punc-122::GFP];xyhEx1008[Pges-1::pmk-2 RNAi, Punc-122::dsRed]
PLX1091 sid-1(qt9);xyhEx498[Psri-36::GFP, Psrh-142::NLS::mCherry, Punc-122::GFP];xyhEx1008[Pges-1::pmk-2 RNAi, Punc-122::dsRed]
PLX1009 xyhEx498[Psri-36::GFP, Psrh-142::NLS::mCherry, Punc-122::GFP];xyhEx1008[Pges-1::daf-16aAM::EGFP, Punc-122::dsRed]
PLX980 xyhEx827[Pges-1::aak-2 RNAi, Pmyo-3::mStrawberry]
PLX1093 sid-1(qt9);xyhEx827[Pges-1::aak-2 RNAi, Pmyo-3::mStrawberry]
PLX981 xyhEx802[Pges-1::daf-16 RNAi, Pmyo-3::mStrawberry]
PLX1094 sid-1(qt9);xyhEx802[Pges-1::daf-16 RNAi, Pmyo-3::mStrawberry]
PLX1010 xyhEx1010[Pges-1::aak-2 RNAi, Pges-1::daf-16 RNAi, Pmyo-3::mStrawberry]
PLX1092 sid-1(qt9);xyhEx1010[Pges-1::aak-2 RNAi, Pges-1::daf-16 RNAi, Pmyo-3::mStrawberry]
PLX982 xyhEx709[Pges-1::pmk-2 RNAi, Pmyo-3::mStrawberry]
PLX1095 sid-1(qt9);xyhEx709[Pges-1::pmk-2 RNAi, Pmyo-3::mStrawberry]
PLX983 xyhEx940[Pges-1:daf-16aAM::EGFP, Pmyo-3::mStrawberry]
PLX984 xyhEx941[Pges-1)::pmk-2 RNAi, Pges-1::daf-16aAM::EGFP, Pmyo-3::mStrawberry]
PLX1096 sid-1(qt9);xyhEx941[Pges-1::pmk-2 RNAi, Pges-1:daf-16 cDNAaAM::EGFP, Pmyo-3::mStrawberry]
PLX781 odr-7(ky4);xyhEx580[Pges-1::hid-1 RNAi, Pmyo-2::mStrawberry];xyhEx446[Psrh-142::HisCl1::SL2::GCaMP6s, Punc-122::GFP]
PLX1070 sid-1(qt9);odr-7(ky4);xyhEx580[Pges-1::hid-1 RNAi, Pmyo-2::mStrawberry];xyhEx446[Psrh-142::HisCl1::SL2::GCaMP6, Punc-122::GFP]

PLX721 egl-3(tm1377);odr-7(ky4);xyhEx446[Psrh-142::HisCl1::SL2::GCaMP6s, Punc-122::GFP]
PLX699 odr-7(ky4);xyhEx446[Psrh-142::HisCl1::SL2::GCaMP6s, Punc-122::GFP];xyhEx699[Psrh-142::daf-2 RNAi, Pmyo-3::mStrawberry]
PLX733 odr-7(ky4);xyhEx446[Psrh-142::HisCl1::SL2::GCaMP6s, Punc-122::GFP];xyhEx733[Pges-1::ins-37 RNAi, Pmyo-3::mStrawberry]
PLX1068 sid-1(qt9);odr-7(ky4);xyhEx446[Psrh-142::HisCl1::SL2::GCaMP6, Punc-122::GFP];xyhEx733[Pges-1::ins-37 RNAi, Pmyo-3::mStrawberry]
PLX712 odr-7(ky4);xyhEx446[Psrh-142::HisCl1::SL2::GCaMP6s, Punc-122::GFP];xyhEx712[Pges-1::ins-7 RNAi, Pmyo-3::mStrawberry]
PLX1069 sid-1(qt9);odr-7(ky4);xyhEx446[Psrh-142::HisCl1::SL2::GCaMP6, Punc-122::GFP];xyhEx712[Pges-1::ins-7 RNAi, Pmyo-3::mStrawberry]
ZC1436 yxIs13[Pins-7::GFP, Punc-122::dsRed]
PLX1072 sid-1(qt9);yxIs13[Pins-7::GFP, Punc-122::dsRed]
PLX1011 yxIs13[Pins-7::GFP, Punc-122::dsRed];xyhEx709[Pges-1::pmk-2 RNAi, Pmyo-3::mStrawberry]
PLX1071 sid-1(qt9);yxIs13[Pins-7::GFP, Punc-122::dsRed];xyhEx709[Pges-1::pmk-2 RNAi, Pmyo-3::mStrawberry]
PLX1012 yxIs13[Pins-7::GFP, Punc-122::dsRed];xyhEx940[Pges-1::daf-16aAM::EGFP, Pmyo-3::mStrawberry]
PLX1013 yxIs13[Pins-7::GFP, Punc-122::dsRed];xyhEx941[Pges-1::pmk-2 RNAi, Pges-1::daf-16aAM::EGFP, Pmyo-3::mStrawberry]
PLX1074 sid-1(qt9);yxIs13[Pins-7::GFP, Punc-122::dsRed];xyhEx941[Pges-1::pmk-2 RNAi, Pges-1::daf-16 cDNAaAM::EGFP, Pmyo-3::mStrawberry]
PLX1087 sid-1(qt9);yxIs13[Pins-7::GFP(70ng/ul), Punc-122::dsRed];xyhEx1087[Pges-1::aak-2 RNAi, Pmyo-2::mStrawberry]
PLX783 xyhEx783[Pins-37::GFP, Pmyo-2::mStrawberry]
PLX1075 sid-1(qt9);xyhEx783[Pins-37::GFP, Pmyo-2::mStrawberry]
PLX737 xyhEx783[Pins-37::GFP, Pmyo-2::mStrawberry];xyhEx827[Pges-1::aak-2 RNAi, Pmyo-3::mStrawberry]
PLX1076 sid-1(qt9);xyhEx783[Pins-37::GFP, Pmyo-2::mStrawberry];xyhEx827[Pges-1::aak-2 RNAi, Pmyo-3::mStrawberry]
PLX738 xyhEx783[Pins-37::GFP, Pmyo-2::mStrawberry];xyhEx802[Pges-1::daf-16 RNAi, Pmyo-3::mStrawberry]
PLX1077 sid-1(qt9);xyhEx783[Pins-37::GFP, Pmyo-2::mStrawberry];xyhEx802[Pges-1::daf-16 RNAi, Pmyo-3::mStrawberry]
PLX739 xyhEx783[Pins-37::GFP, Pmyo-2::mStrawberry];xyhEx1010[Pges-1::aak-2 RNAi, Pges-1::daf-16 RNAi, Pmyo-3::mStrawberry]
PLX1078 sid-1(qt9);xyhEx783[Pins-37::GFP, Pmyo-2::mStrawberry];xyhEx1010[Pges-1::aak-2 RNAi, Pges-1::daf-16 RNAi, Pmyo-3::mStrawberry]
PLX1139 sid-1(qt9);xyhEx783[Pins-37::GFP, Pmyo-2::mStrawberry];xyhEx1139[Pges-1::pmk-2 RNAi, Pmyo-3::mStrawberry]
PLX1014 xyhEx498[Psri-36::GFP, Psrh-142::NLS::mCherry, Punc-122::GFP];xyhEx1014[Pges-1::ins-37 RNAi, Punc-122::dsRed]
PLX1015 xyhEx498[Psri-36::GFP, Psrh-142::NLS::mCherry, Punc-122::GFP];xyhEx1015[Psrh-142::daf-2 RNAi, Punc-122::dsRed]
PLX1016 xyhEx498[Psri-36::GFP, Psrh-142::NLS::mCherry, Punc-122::GFP];xyhEx1016[Psrh-142::daf-2 RNAi, Pges-1::ins-37 RNAi, Punc-122::dsRed]
PLX1017 xyhEx498[Psri-36::GFP, Psrh-142::NLS::mCherry, Punc-122::GFP];xyhEx1017[Psrh-142::daf-16 RNAi, Punc-122::dsRed]
PLX1018 xyhEx498[Psri-36::GFP, Psrh-142::NLS::mCherry, Punc-122::GFP];xyhEx1018[Psrh-142::daf-16aAM, Punc-122::dsRed]
PLX1019 xyhEx498[Psri-36::GFP, Psrh-142::NLS::mCherry, Punc-122::GFP];xyhEx1019[Pges-1::ins-7 RNAi, Punc-122::dsRed]
PLX985 xyhEx712[Pges-1::ins-7 RNAi, Pmyo-3::mStrawberry]
PLX317 xyhEx500[Pges-1::ins-37 RNAi, Pmyo-3::mStrawberry]
PLX587 xyhEx587[Psrh-142::daf-2 RNAi, Pmyo-3::mStrawberry]
PLX992 xyhEx992[Psrh-142::daf-2 RNAi, Pges-1::ins-37 RNAi, Pmyo-3::mStrawberry]
PLX993 xyhEx993[Psrh-142::daf-2 RNAi, Pges-1::ins-7 RNAi, Pmyo-3::mStrawberry]
PLX1020 xyhEx823[Psrh-142::daf-16 RNAi, Pmyo-3::mStrawberry]
PLX1021 xyhEx1021[Psrh-142::daf-16aAM, Pmyo-3::mStrawberry]
PLX585 xyhIs585[Pgcy-28::HisCl::SL2::GCaMP6s, lin-15(+)]
PLX367 xyhEx367[Pttx-3::HisCl1::SL2::GCaMP6s, Pmyo-3::mStrawberry]
PLX420 xyhIs585[Pgcy-28::HisCl::SL2::GCaMP6s, lin-15(+)];xyhEx367[Pttx-3::HisCl1::SL2::GCaMP6s, Pmyo-3::mStrawberry]
PLX508 xyhIs508[Pnpr-9::HisCl1::SL2::mStrawberry, Pnpr-9::GCaMP6s, Punc-122::dsRed]
PLX448 xyhEx448[Podr-2::HisCl::SL2::GCaMP6s, Punc-122::dsRed]
PLX1022 xyhEx1022[Pser-2prom2::HisCl::SL2::GCaMP6s, Pmyo-3::mStrawberry]
PLX494 xyhIs494[Pflp-8::HisCl::SL2::GCaMP6s, Punc-122::dsRed]
PLX1023 xyhIs494[Pflp-8::HisCl::SL2::GCaMP6s, Punc-122::dsRed];xyhIs508[Pnpr-9::HisCl1::SL2::mStrawberry, Pnpr-9::GCaMP6s, Punc-122::dsRed]
PLX470 xyhIs470[Pgcy-36::HisCl1::SL2::GCaMP6, Punc-122::dsRed]
PLX411 xyhEx411[Pglr-3::HisCl1::SL2::GCaMP6s, Punc-122::dsRed]
PLX1024 odr-7(ky4);xyhEx446[Psrh-142::HisCl1::SL2::GCaMP6s, Punc-122::GFP];xyhIs508[Pnpr-9::HisCl1::SL2::mStrawberry, Pnpr-9::GCaMP6s, Punc-122::dsRed]
PLX1025 odr-7(ky4);xyhEx446[Psrh-142::HisCl1::SL2::GCaMP6s, Punc-122::GFP];xyhIs494[Pflp-8::HisCl::SL2::GCaMP6s, Punc-122::dsRed]
PLX1026 odr-7(ky4);xyhEx446[Psrh-142::HisCl1::SL2::GCaMP6s, Punc-122::GFP];xyhIs585[Pgcy-28::HisCl::SL2::GCaMP6s, lin-15(+)]
PLX1027 odr-7(ky4);xyhEx446[Psrh-142::HisCl1::SL2::GCaMP6s, Punc-122::GFP];xyhEx367[Pttx-3::HisCl1::SL2::GCaMP6s, Pmyo-3::mStrawberry]
PLX1028 ser-4(ok512);xyhEx1028[Pnpr-9::ser-4 cDNA, Pmyo-3::mStrawberry]
PLX1029 lgc-50(tm3712);xyhEx1029[Pflp-8::lgc-50 cDNA::SL2::GFP, Pmyo-3::mStrawberry]
PLX676 mod-1(ok103);xyhEx676[Pgcy-28::mod-1 gDNA, Pttx-3::mod-1 gDNA, Pmyo-3::mStrawberry]
PLX831 xyhIs508[Pnpr-9::HisCl1::SL2::mStrawberry, Pnpr-9::GCaMP6s, Punc-122::dsRed];xyhEx831[Pnpr-9::ser-4 RNAi, Pmyo-3::mStrawberry]
PLX943 xyhEx943[Pflp-8::lgc-50 RNAi, Pflp-8::HisCl::SL2::GCaMP6s, Pmyo-2::mStrawberry]
PLX835 xyhIs585[Pgcy-28::HisCl::SL2::GCaMP6s, lin-15(+)];xyhEx835[Pgcy-28::mod-1 RNAi, Pmyo-2::mStrawberry]
PLX949 xyhEx949[Pttx-3::mod-1 RNAi, Pttx-3::HisCl1::SL2::GCaMP6s, Pmyo-2::mStrawberry]
PLX468 xyhIs461[Pceh-2::HisCl1::SL2::GCaMP6s, Punc-122::dsRed]
PLX837 xyhEx837[Pclh-3::HisCl::SL2::GCaMP6s, Pmyo-2::mStrawberry]
CX17462 kyEx6119[Pgpa-6::GCaMP6f;Punc-122::dsRed]
JKG1058 gonEx113[Psra-6::GCaMP6s, Psra-6::SL2::mCherry]
PLX646 xyhIs646[Podr-7::HisCl1::SL2::GCaMP6s, Pmyo-2::mStrawberry]

PLX851 xyhEx851[Psra-6::sri-36 cDNA, Pmyo-3::mStrawberry];gonEx113[Psra-6::GCaMP6s, Psra-6::SL2::mCherry]
PLX959 odr-7(ky4);xyhEx959[Psra-6::sri-36 cDNA, Pmyo-3::mStrawberry]
VP303 rde-1(ne219);kbIs7[nhx-2p::rde-1, rol-6(su1006)]
PLX802 odr-7(ky4);xyhEx802[Pges-1::daf-16 RNAi, Pmyo-3::mStrawberry];xyhEx446[Psrh-142::HisCl1::SL2::GCaMP6s, Punc-122::GFP]
PLX829 odr-7(ky4);xyhEx829[Psrh-142::hsf-1 RNAi, Pmyo-3::mStrawberry];xyhEx446[Psrh-142::HisCl1::SL2::GCaMP6s, Punc-122::GFP]
PLX325 odr-7(ky4);xyhEx325[Psrh-142::daf-12 RNAi, Pmyo-3::mStrawberry];xyhEx446[Psrh-142::HisCl1::SL2::GCaMP6s, Punc-122::GFP]
PLX364 odr-7(ky4);xyhEx364[Psrh-142::pha-4 RNAi, Pmyo-3::mStrawberry];xyhEx446[Psrh-142::HisCl1::SL2::GCaMP6s, Punc-122::GFP]
PLX846 odr-7(ky4);xyhEx846[Psrh-142::skn-1 RNAi, Pmyo-3::mStrawberry];xyhEx446[Psrh-142::HisCl1::SL2::GCaMP6s, Punc-122::GFP]
PLX590 odr-7(ky4);xyhEx446[Psrh-142::HisCl1::SL2::GCaMP6s, Punc-122::GFP];xyhEx590[Psrh-142::pqm-1 RNAi, Pmyo-2::mStrawberry]
PLX1030 odr-7(ky4);xyhEx446[Psrh-142::HisCl1::SL2::GCaMP6s, Punc-122::GFP];xyhIs508[Pnpr-9::HisCl1::SL2::mStrawberry, Pnpr-9::GCaMP6s, Punc-122::dsRed];xyhIs494[Pflp-8::HisCl::SL2::GCaMP6s, Punc-122::dsRed]
PLX1031 odr-7(ky4);xyhEx446[Psrh-142::HisCl1::SL2::GCaMP6s, Punc-122::GFP];xyhIs585[Pgcy-28::HisCl::SL2::GCaMP6s, lin-15(+)];xyhEx367[Pttx-3::HisCl1::SL2::GCaMP6s, Pmyo-3::mStrawberry]
PLX766 xyhEx766[Psrh-142::TrpV1, Pmyo-3::mStrawberry]
PLX1032 xyhEx766[Psrh-142::TrpV1, Pmyo-3::mStrawberry];xyhEx1032[Pgcy-28::mod-1 RNAi, Pttx-3::mod-1 RNAi, Pmyo-2::mStrawberry]
PLX1033 xyhEx446[Psrh-142::HisCl1::SL2::GCaMP6, Punc-122::GFP];xyhEx1033[Pnpr-9::ser-4 RNAi, Pflp-8::lgc-50 RNAi, Pmyo-3::mStrawberry]
PLX1036 odr-7(ky4);xyhEx1036[Psrh-142::SNB-1::Linker::pHluorin, Psrh-142::mStrawberry, Punc-122::GFP]
PLX1138 sri-36(plc921);;odr-7(ky4);xyhEx1036[Psrh-142::SNB-1::Linker::pHluorin, Psrh-142::mStrawberry, Punc-122::GFP]
PLX1108 sri-36(plc921);odr-7(ky4);yhEx1104[Psrh-142::sri-36 gDNA::mStrawberry (5ng/ul), Punc-122::dsRed];xyhEx1036[Psrh-142::SNB-1::Linker::pHluorin, Psrh-142::mStrawberry, Punc-122::GFP]
PLX1109 sri-36(plc921);odr-7(ky4);yhEx1104[Psrh-142::sri-36 gDNA::mStrawberry (75ng/ul), Punc-122::dsRed];xyhEx1036[Psrh-142::SNB-1::Linker::pHluorin, Psrh-142::mStrawberry, Punc-122::GFP]

Wild animals
The study did not involve wild animals.

Reporting on sex
All experiments were performed using young adult hermaphrodites.

Field-collected samples
The study did not involve samples collected from the field.

Ethics oversight
The study uses the nematode C. elegans, which does not require Ethical approval.

Note that full information on the approval of the study protocol must also be provided in the manuscript.

Plants

Seed stocks
not applicable

Novel plant genotypes
not applicable

Authentication
not applicable
